# Acute Complete Foot Drop Caused by Intraneural Ganglion Cyst without a Prior Traumatic Event

**DOI:** 10.1155/2020/1904595

**Published:** 2020-03-04

**Authors:** Stavros Stamiris, Dimitrios Stamiris, Athanasios Sarridimitriou, Elissavet Anestiadou, Christos Karampalis, Vasileios Vrangalas

**Affiliations:** ^1^Orthopaedic Department, Papageorgiou General Hospital, Ring Road West 56403, Nea Efkarpia, Thessaloniki, Greece; ^2^Orthopaedic Department, 424 General Military Hospital, Ring Road West 56429, Nea Efkarpia, Thessaloniki, Greece; ^3^Faculty of Medicine, Aristotle University, 54124 Thessaloniki, Greece

## Abstract

Intraneural ganglion cysts are benign soft-tissue masses located in the epineurium of peripheral nerves. They originate from nearby joint connections via articular branches. Traumatic events seem to play a role in their pathogenesis as well. Clinical manifestations include pain over the area of the cyst, palpable tender mass, hypoesthesia, and muscle weakness depending on the affected nerve. Our case highlights an uncommon clinical manifestation of this entity with acute foot drop, as the primary symptom, without any previous traumatic event, enriching by this way the current diagnostic thinking process of clinical physicians. We report a case of a 42-year-old military officer who presented to our emergency department with acute foot drop that appeared during a march. Initially, the common peroneal palsy was misdiagnosed as L5-S1 disc herniation, but investigation with lumbar MRI scan led to rejection of our primary diagnosis. After performing EMG of the lower extremity and knee MRI, an intraneural ganglion cyst of the common peroneal nerve was diagnosed. Patient was treated with surgical decompression of the cyst, followed by ligation and complete resection of the articular branch, as well as disarticulation of the superior tibiofibular joint. At a twelve-month follow-up, the patient showed significant functional recovery. This is, to the best of our knowledge, the first case of intraneural ganglion cyst manifested with an acute complete foot drop without a clear prior traumatic event. We underline the need for a high index of suspicion when dealing with cases of acute peroneal palsy without any accompanying symptoms.

## 1. Introduction

Intraneural ganglion cysts represent rare benign cystic lesions formed within the epineurium of peripheral nerves, near joints. The most common site affected is the common peroneal nerve and its branches, while similar cysts of the ulnar, sciatic, and tibial nerves have also been reported [1, 2].

Patients usually experience pain, numbness, hypoesthesia, and muscle weakness along the distribution of the affected nerve. Positive Tinel's sign and a palpable tender mass around the area of the ganglion cyst are also common. The onset of symptoms can be gradual or, less commonly, acute, usually exacerbated by a previous traumatic event [3].

The exact etiology of this condition remains unknown. Spinner et al. proposed the unifying articular theory, which highlights the key role of the articular branch in the pathogenesis of an endoneural ganglion cyst [4]. According to their theory, the origin of the cyst is the nearby joint. Through a capsular defect (traumatic or degenerative), synovial fluid enters the articular branch via a one-way valve mechanism and tracks proximally, dissecting the epineurium, until it reaches the main trunk of the nerve [4].

## 2. Case Presentation

This study was conducted in accordance with the ethical standards of the institutional review board of our hospital and with the 1964 Helsinki Declaration and its later amendments or comparable ethical standards [5].

A 42-year-old male presented to the emergency department of our military hospital with reported mild low back pain, associated with numbness in his right leg below the knee, and ipsilateral complete foot drop that occurred during a military march. The onset of neurological symptoms was acute, without any previous symptoms, reaching immediately its full intensity and was perceived by the patient as dragging of the foot. The patient had no history of lumbar spine disease. No traumatic event was reported during the march.

Physical examination of the right lower extremity showed negative Lasegue's sign and straight leg raise test and hypoesthesia in the lateral aspect of the leg and dorsal aspect of the foot. Foot dorsiflexion and eversion and large toe dorsiflexion were severely impaired (tibialis anterior, extensor hallucis longus, extensor digitorum longus, and peroneus muscle strength assessment revealed a grade of 1/5 in the MRC scale). There were no clinical signs of muscle atrophy on the anterior and lateral compartments of the leg. Physical examination of the contralateral lower extremity was normal. Knee and lumbar X-rays were normal.

**Figure 1:**
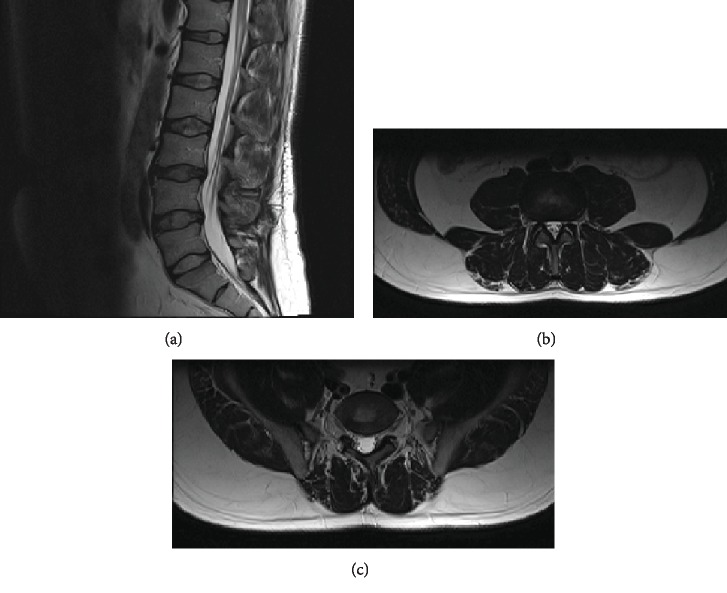
(a) Sagittal T2 lumbar spine MRI showing multiple disc bulges at L3/L4 and L4/L5. (b) Axial T2 at the L4/L5 level showing a mild disc bulge narrowing the right nerve root foramen. (c) Axial T2 at the L5/S1 level showing mild right paracentral disc bulge.

Our initial therapeutic approach included rest, NSAIDs, painkillers, and corticosteroid intramuscular injections. The patient was admitted to our clinic under observation status, and lumbar spine MRI scan was ordered; however, results did not correspond to the patient's symptomatology (Figure 1). Moreover, the patient's blood tests, including inflammatory markers (WBC, CRP, and ESR), were normal.

**Figure 2:**
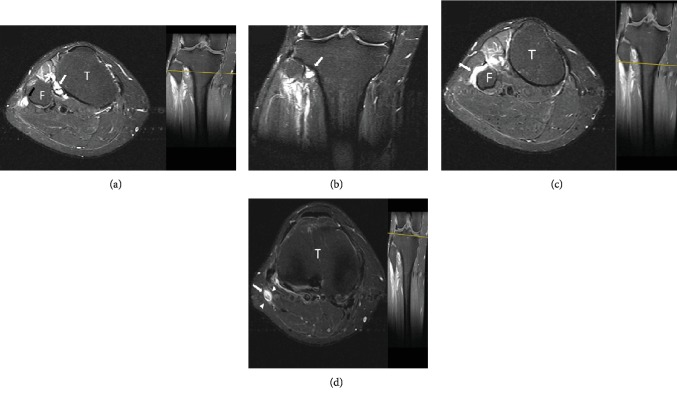
T2-weighted MR axial (a) and coronal (b) images of the knee at the level of the tibiofibular joint. White arrows point to the ganglion cyst extending from the STF joint, and the black arrow points to the ascending oedematous CPN. T2-weighted axial image at the level of the fibular neck (c). A horizontal, linear area of increased T2 signal along the course of the nerve represents the extension of the ING along the transverse limb of the peroneal nerve articular branch (white arrow). T2-weighted axial image above the level of the fibular neck (d) showing an intraneural ganglion cyst (white arrow) in which the tibial and peroneal divisions are separately contained (arrowheads) T: tibia; F: fibula.

Following negative lumbar spine MRI results, a second more thorough physical examination was conducted, which revealed positive Tinel's sign in the area of the fibular neck, shifting diagnostic thinking process towards peripheral neuropathy. Subsequent EMG and NCS showed decreased conduction velocity and amplitude in the common peroneal nerve around the area of the popliteal fossa. Furthermore, knee MRI showed a multilobulated lesion in the area around the fibular head, in close proximity to the common peroneal nerve (Figure 2).

**Figure 3:**
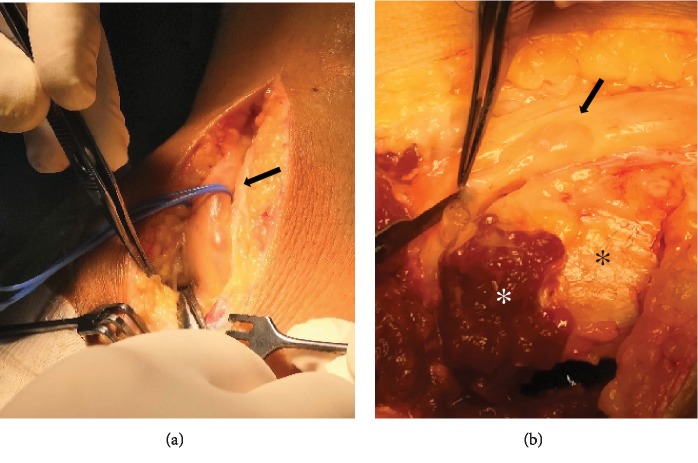
Photographs demonstrating the oedematous common peroneal nerve (black arrow) intraoperatively. In (b), an incision was made in the epineurium to enable the evacuation of the mucoid content and the decompression of the nerve. Fibular head (black asterisk) and the dissected peroneus longus muscle (white asterisk) can also be seen.

**Figure 4:**
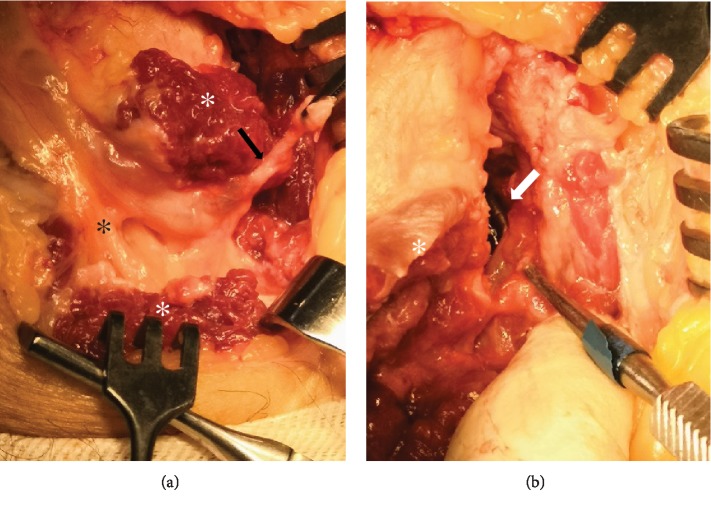
Photographs illustrating the articular branch ((a) black arrow) to the superior tibiofibular joint ((b) white arrow). Transection of this branch after ligation alongside disarticulation of the superior TF joint is essential to prevent recurrence. In (a), deep peroneal nerve (black asterisk) and the peroneus longus muscle (white asterisk) can also be recognized. The peroneus longus was dissected to allow better view of the deep and superficial peroneal nerves, as well as the peroneal articular branch.

Surgical exploration under loop magnification was decided. The common peroneal nerve was identified and followed to its bifurcation. Common and deep peroneal nerves appeared oedematous (Figure 3). The articular branch was also identified and surgically prepared from its origin near deep peroneal nerve bifurcation, up to the superior tibiofibular joint. The superior tibiofibular joint was disarticulated, and the articular branch was ligated and transected (Figure 4). A small anatomic specimen of the peroneal articular branch was sent for histologic examination. An incision was made to the epineurium of the common peroneal nerve and mucoid material was evacuated from the ganglion cyst (Figure 3).

**Figure 5:**
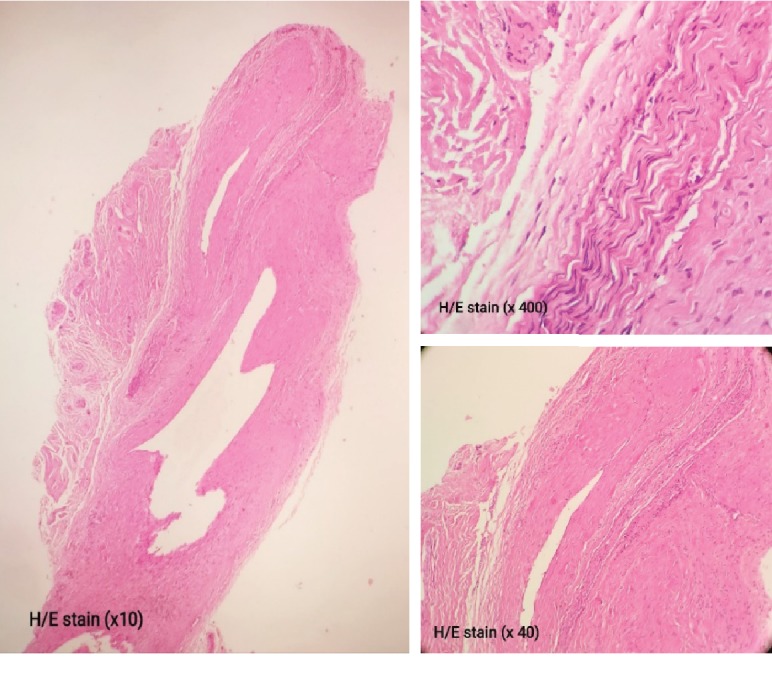
Ganglion of nerve sheath with myxoid change and cystic degeneration seen in the connective tissue of the nerve (H&E ×10, ×40, and ×400).

**Figure 6:**
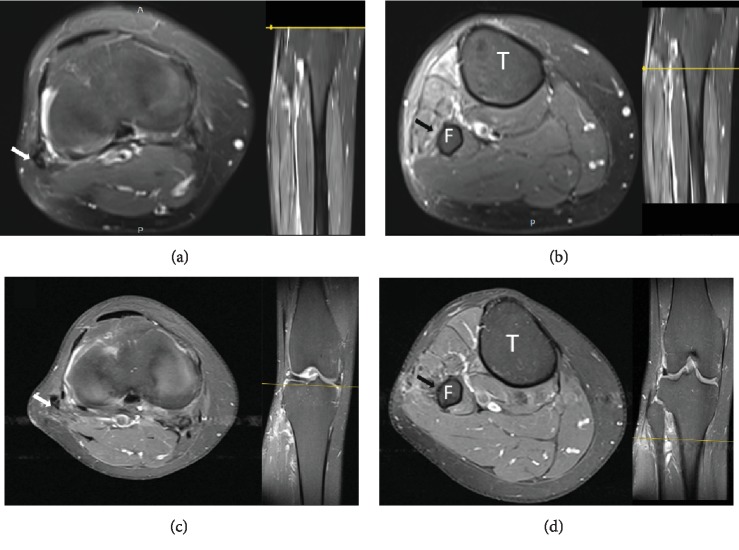
T2-weighted MR axial images of the knee at 6 months (a and b) and 10 months (c and d) postoperative in levels corresponding to preoperative views. a and c are taken at a level above the fibular head. The absence of a high signal is noted in CPN (white arrow). b and d are taken at the level in which a “transverse limb sign” was observed in preoperative MRI (black arrow). T: tibia; F: fibula.

Postoperatively, the patient was treated with a foot drop polyethylene splint and physiotherapy. Histological examination confirmed our diagnosis of an endoneural ganglion cyst (Figure 5). At a 3-month follow-up after the surgical decompression, the patient showed clinical (tibialis anterior 4/5, extensor hallucis longus 2/5, extensor digitorum longus 3/5, and peroneus muscles 4/5 in the MRC scale for muscle strength and complete return of sensation to the lateral aspect of the leg and dorsal aspect of the foot) and EMG evidence of recovery. Subsequent knee MRI showed no signs of recurrence (Figure 6). At the last follow-up, 12 months after surgery, clinical examination revealed functional recovery. Following the aforementioned findings, the patient was allowed to return to his military duties.

## 3. Discussion

Intraneural ganglion cyst cases reported in the literature are rare. The most frequent site of occurrence is reported to be the common peroneal nerve, followed by the ulnar and tibial nerves [6]. Although several theories have been proposed to interpret this pathology (recurrent trauma [3], intraneural hemorrhage [7], mucoid degeneration [8], and de novo formation from hamartomatous cell rests [9]), the articular theory, described by Spinner et al., is the most widely accepted. According to this theory, endoneural ganglion cysts originate from nearby joints (in the case of our patient, from the superior tibiofibular joint). Through a capsular defect, joint fluid exits via a one-way mechanism and tracks along the epineurium of the innervating articular branch following the path of least resistance. In intraneural ganglion cysts of the common peroneal nerve, fluid originates from the superior tibiofibular joint [4]. In the present case, we were able to identify the articular branch during the surgery and resect it. Furthermore, Spinner et al. proposed dynamic aspects of cyst formation, according to which the various patterns of ascent, crossover, and descent down terminal nerve branches are attributed to intra-articular pressure fluctuation and dynamic pressure fluxes [10]. More recent literature has incorporated the direct or indirect injury of the joint as a key component in the pathogenesis of an intraneural ganglion cyst, adding it up to the articular theory [11, 12].

Common symptomatology of an endoneural ganglion cyst of the common peroneal nerve includes pain over the fibular head, with or without swelling, positive Tinel's sign, and paresthesia over the lateral surface of the tibia and dorsum of the foot. Some patients may present with gradual or acute weakness of the muscles located in the anterior and lateral compartments of the leg. Muscle denervation and atrophy have also been described [3, 13–16]. In our case, the patient presented with an acute painless foot drop that occurred during a military march and developed in a short period of time with no obvious history of trauma. To the best of our knowledge, this is the first reported case of an intraneural ganglion cyst resulting in an acute foot drop without a prior traumatic event. Additionally, a foot drop as the primary symptom is an underreported manifestation of an intraneural ganglion cyst in the literature [14, 17]. Coexisting mild lumbar pain at first led us to a wrong initial assessment.

MRI and/or ultrasound serve as useful tools of the diagnostic process. On ultrasonography, an endoneural ganglion cyst appears as a large well-circumscribed hypoechogenic lesion [18], while on MRI it appears as a multilobulated lesion with low signal intensity on T1-weighted images and high signal on T2-weighted images, oriented longitudinally along the course of the affected nerve. Furthermore, muscle denervation oedema can be seen on T2-weighted images as hyperintensity. Muscle atrophy is also characterized as hyperintensity on T1-weighted images [19]. Recognition of the articular connection is a possible finding, but it is not always easily detected on MRI. Spinner et al. demonstrated three reproducible MRI features that can provide aid in identifying the joint connection (tail sign) and differentiating between intraneural and extraneural ganglion cysts (transverse limb sign, signet ring sign) [20].

Ultrasonography is less time consuming than MRI and may be of value when guided percutaneous aspiration is decided, but it fails to illustrate the relation of the cyst with the neighboring anatomical structures as well as the articular connection and, unless a specialized radiologist in musculoskeletal ultrasonography is available or MRI is contraindicated, intraneural ganglion cysts are best imaged with magnetic resonance [21].

Standard treatment of intraneural ganglion cysts is surgical excision of the ganglion and nerve decompression. An alternative minimally invasive treatment is decompression by percutaneous aspiration of the ganglion under ultrasound guidance, with or without corticosteroid injection [13]. Both these methods have reported high recurrence rates (38% and 50-70%, respectively). The best results in terms of recurrence are obtained with surgical decompression and complete removal of the cyst followed by ligation and complete resection of the articular branch as well as disarticulation of the involved joint at the cost of higher risk of iatrogenic nerve injury [6, 21]. Desy et al., in their review, advocated the simple incision and decompression of the cyst followed by STFJ resection and ligation of the articular branch [6]. Our patient was treated with surgical decompression via a simple incision followed by ligation and transection of the articular branch as well as disarticulation of the STFJ. At 12 months postoperatively, no recurrence was observed and the patient regained functional recovery.

This article demonstrates a rare case of an acute foot drop caused by an intraneural ganglion cyst. To the best of our knowledge, this is the first case of an intraneural ganglion cyst resulting in an acute foot drop without a prior traumatic event. Our hypothesis is that of an existing asymptomatic intraneural ganglion cyst that deteriorated past the asymptomatic threshold point during the walk, possibly via an indirect mechanism, as proposed by Spinner et al. [11]. Although a clear traumatic event was not reported, it is possible that a minor ankle torsional strain was transmitted through interosseous membrane to STFJ, aggravating the preexisting ganglion cyst [11]. Furthermore, this article provides additional support for the unifying articular theory, as proposed by Spinner et al. [4].

## 4. Conclusion

Intraneural ganglion cysts, although infrequent, are well established in the literature and should be considered in the differential diagnosis of peripheral mononeuropathy, in order to avoid diagnostic pitfalls. Early diagnosis and surgical treatment with open decompression and concurrent address of the articular branch are of paramount importance to obtain positive outcome and minimize recurrence risk.

## Abbreviations

EMG: Electromyogram test
NCS: Nerve conduction study
NSAID: Nonsteroid anti-inflammatory drugs
MRI: Magnetic resonance imaging
WBC: White blood cell
CRP: C-reactive protein
ESR: Erythrocyte sedimentation rate
MRC: Medical Research Council
STFJ: Superior tibiofibular joint
CPN: Common peroneal nerve.
